# Pharmacological ascorbate inhibits pancreatic cancer metastases via a peroxide-mediated mechanism

**DOI:** 10.1038/s41598-020-74806-2

**Published:** 2020-10-19

**Authors:** Brianne R. O’Leary, Matthew S. Alexander, Juan Du, Devon L. Moose, Michael D. Henry, Joseph J. Cullen

**Affiliations:** 1 Department of Surgery, The University of Iowa Carver College of Medicine, Iowa City, IA USA; 2 Department of Molecular Physiology and Biophsics, The University of Iowa Carver College of Medicine, Iowa City, IA USA; 3 The Holden Comprehensive Cancer Center, University of Iowa Hospitals and Clinics, The University of Iowa Carver College of Medicine, 1528 JCP, 200 Hawkins Drive, Iowa City, IA 52242 USA; 4 Department of Pathology, The University of Iowa Carver College of Medicine, Iowa City, IA USA; 5 Department of Urology, The University of Iowa Carver College of Medicine, Iowa City, IA USA; 6 Free Radical and Radiation Biology Program, Department of Radiation Oncology, The University of Iowa Carver College of Medicine, Iowa City, IA USA

**Keywords:** Cancer, Medical research, Oncology

## Abstract

Pharmacological ascorbate (P-AscH^−^, high-dose, intravenous vitamin C) is cytotoxic to tumor cells in doses achievable in humans. Phase I studies in pancreatic cancer (PDAC) utilizing P-AscH^−^ have demonstrated increases in progression free survival, suggesting a reduction in metastatic disease burden. The purpose of this study was to determine the effects of P-AscH^−^ on metastatic PDAC. Several in vitro and in vivo mechanisms involved in PDAC metastases were investigated following treatment with P-AscH^−^. Serum from PDAC patients in clinical trials with P-AscH^−^ were tested for the presence and quantity of circulating tumor cell-derived nucleases. P-AscH^−^ inhibited invasion, basement membrane degradation, decreased matrix metalloproteinase expression, as well as clonogenic survival and viability during exposure to fluid shear stress. In vivo*,* P-AscH^−^ significantly decreased formation of ascites, tumor burden over time, circulating tumor cells, and hepatic metastases. Both in vitro and in vivo findings were reversed with the addition of catalase suggesting that the effect of P-AscH^−^ on metastatic disease is mediated by hydrogen peroxide. Finally, P-AscH^−^ decreased CTC-derived nucleases in subjects with stage IV PDAC in a phase I clinical trial. We conclude that P-AscH^−^ attenuates the metastatic potential of PDAC and may prove to be effective for treating advanced disease.

## Introduction

Pancreatic ductal adenocarcinoma (PDAC) is the 3rd most common cause of cancer-related death with over 45,750 fatal cases reported annually in the U.S. alone^1^. The incidence of PDAC continues to rise and 5-year survival rates for patients with metastatic disease is still currently below 3%^1^. Systemic chemotherapy provides only temporary benefits in advanced metastatic disease whereas it prolongs survival in the adjuvant setting presumably by targeting microscopic foci of local and distant disease. Clearly, a major research effort is required to better understand the mechanisms regulating metastatic disease in this malignancy.

Previous studies suggest that the primary tumor in PDAC may grow for several years before producing metastases^2^. However, Rhim and colleagues demonstrated that PDAC has the ability to invade and enter the bloodstream very early, even prior to the detection of a primary tumor^3^. Consistent with these latter findings, nearly 75% of patients present with metastatic disease, demonstrating that the tendency for PDAC to progress early and aggressively contributes to the lethal nature of this disease.

Our group and others have demonstrated that high-dose, intravenous, pharmacological ascorbate (P-AscH^−^) is cytotoxic and induces oxidative stress selectively in PDAC cells but not in normal cells^4–6^. P-AscH^−^ cytotoxicity occurs when P-AscH^−^ undergoes auto-oxidation resulting in the generation of hydrogen peroxide (H_2_O_2_). Furthermore, phase I clinical trials have demonstrated P-AscH^−^ to be safe and well tolerated in combination with standard of care chemotherapeutics (gemcitabine + erlotinib, gemcitabine alone, and gemcitabine + radiation) for the treatment of PDAC^6–8^.

We hypothesized that P-AscH^−^ has the potential to inhibit PDAC metastases which is based on three distinct observations. First, previous studies have demonstrated that oxidative stress inhibits distant tumor metastases in an in vivo model^9^. P-AscH^−^, which causes oxidative stress in cancer cells^6^, would lead to an inhibition in PDAC metastases. Second, combining epithelial to mesenchymal transition (EMT) inhibition with chemotherapy reduces chemoresistance in PDAC^10^, again strongly supporting the role of P-AscH^−^ in reducing metastatic disease by inhibiting the EMT process and tumor cell invasion via a potential peroxide-mediated mechanism. Finally, two phase I studies (NCT 01049880 & NCT 01852890)^6,7^ at The University of Iowa demonstrated that patients receiving P-AscH^−^ had increased progression free survival compared to historical controls in both studies, suggesting that P-AscH^−^ can reduce metastatic disease burden in PDAC.

Our current study demonstrates that P-AscH^−^ inhibits invasion and degradation of the basement membrane as well as matrix metalloproteinases associated with metastatic disease progression in metabolically active PDAC cells in vitro*.* In a model relevant to the survival of circulating tumor cells (CTCs)^11^, PDAC cells treated with P-AscH^−^ decreases clonogenic survival along with viability during exposure to fluid shear stress of cells in suspension. Also, P-AscH^−^ decreases CTCs, hepatic metastases, and development of ascites in vivo, which appears to be mediated by peroxide generation. Finally, P-AscH^−^ decreases circulating tumor cell derived nucleases in patients with stage IV PDAC. P-AscH^−^ represents an entirely novel adjuvant to treat PDAC. Recent advances in treatment success have only led to modest improvements, so relatively non-toxic adjuvants (i.e., P-AscH^−^) that could improve outcome and be easily implemented in multi-center trials would be highly significant.

## Results

### P-AscH^−^ inhibition of the invasive phenotype of PDAC is mediated by peroxide

**Figure 1:**
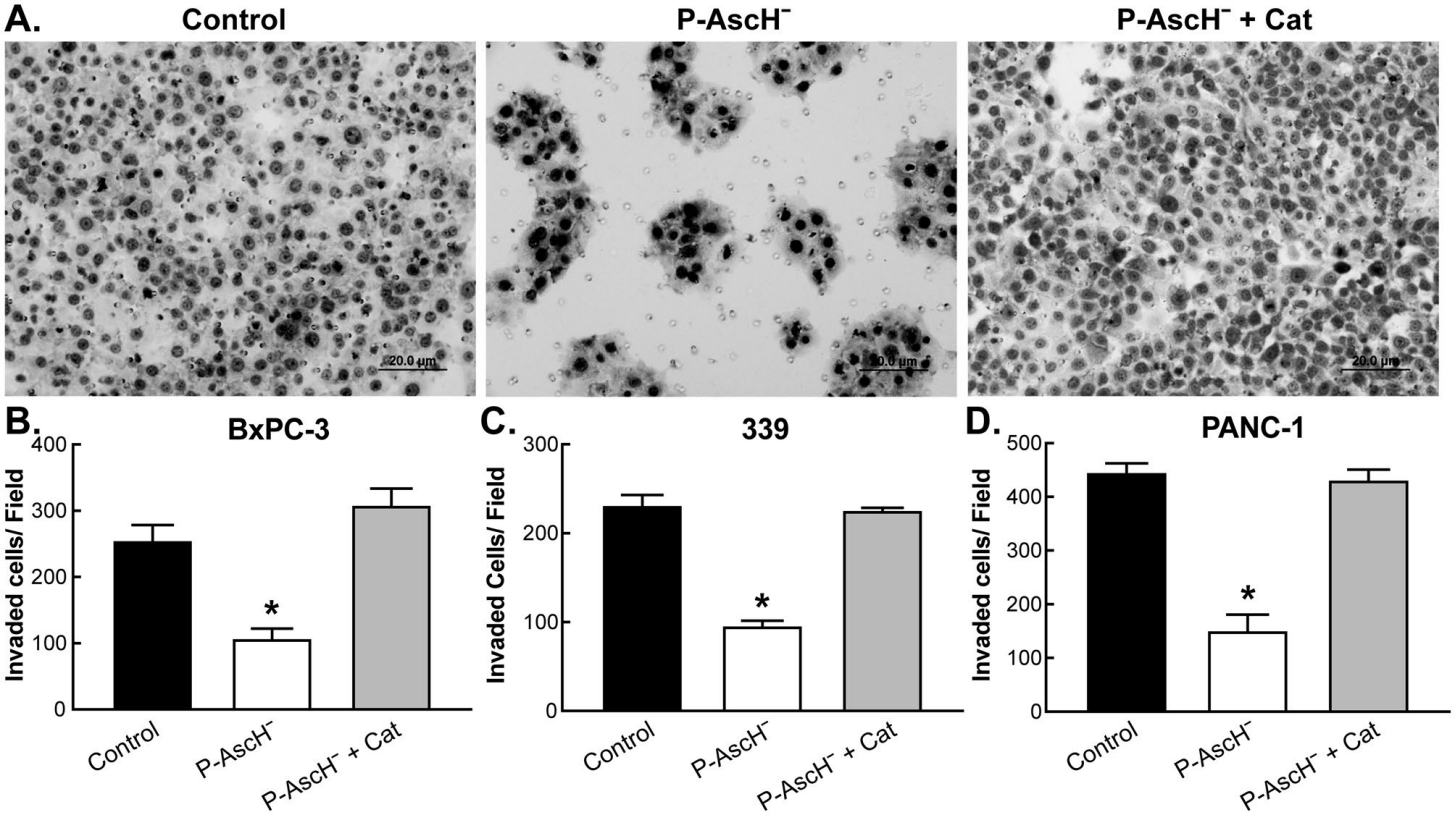
P-AscH^−^ attenuates the invasive phenotype of PDAC in vitro*.* Cells were treated with P-AscH^−^ or P-AscH^−^ + catalase (200 U/mL) for 1 h then seeded at 1–3 × 10^5^ and incubated for 24 (PANC-1) or 48 h (BxPC-3 and 339). Data represent mean of invaded cells/field compared to control ± SE (n = 5, *p < 0.05; one-way ANOVA with Bonferroni’s multiple comparisons). (**A**) Representative invasion images from BxPC-3 PDAC cells in the presence of P-AscH^−^ and/or catalase. (**B**) P-AscH^−^ (2 mM for 1 h) decreased the percentage of invading BxPC-3 cells by 42% (255 +/− 23 cells vs 107 ± 15 cells). (**C**) P-AscH^−^ (4 mM for 1 h) decreased the percentage of invading 339 patient derived PDAC cells by 41.5% (231 ± 11.5 cells vs 96 +/− 6 cells). (**D**) P-AscH^−^ (4 mM for 1 h) decreased the percentage of invading Panc-1 PDAC cells by 34% (455 +/− 16.5 cells vs 151 +/− 30 cells).

To determine the ability for cells to invade through the extracellular matrix (ECM) an invasion assay was performed. Figure 1A–D and Supplemental Figure 1 demonstrate that P-AscH^−^ decreases invasion in the PDAC cells BXPC-3 and PANC-1 as well as the patient derived cell line 339. The decreases in invasion were reversed in each cell line by the addition of catalase (Fig. 1B–D) suggesting that peroxide mediates this effect. Previous studies from our laboratory have demonstrated that PDAC cells are viable at this time point^12,13^, which further supports the hypothesis that P-AscH^−^ induced generation of H_2_O_2_ mediates the inhibition of PDAC invasion as opposed to killing the cells which would indirectly inhibit invasion.

**Figure 2:**
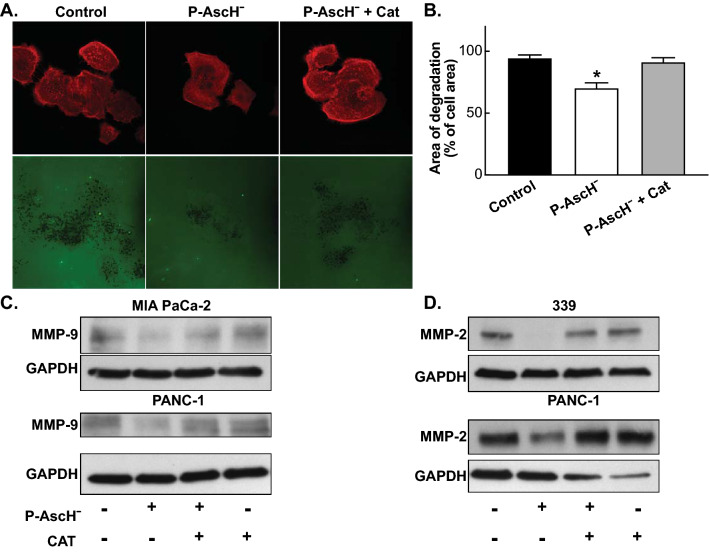
P-AscH^−^ decreases invadopodia-mediated ECM degradation and down regulates MMP-9 and MMP-2 expression. (**A**) P-AscH^−^ attenuates the degradation of fluorescent gelatin matrices by invadopdia of BxPC-3 PDAC cells. BxPC-3 cells were treated with 1 mM ascorbate with or without catalase (200 U/mL) for 1 h. Following treatment, cells were re-plated on coverslips with Oregon Green-conjugated gelatin for 24 h. Cells were fixed, permeabilized, and stained for actin using TRIC-Phalloidin. Fluorescence images were acquired to determine foci of degraded matrix visible as black spots in the bright fluorescent gelatin matrix. (**B**) P-AscH^−^ decreased the area of degradation of BxPC-3 cells compared to control. The addition of catalase reversed the decrease seen with ascorbate. Data represent the mean area of degradation compared to control ± SE (n = 3, *p < 0.05; one-way ANOVA with Bonferroni’s multiple comparisons). (**C**) MMP-9 expression was decreased in MIA PaCa-2 and PANC-1 cells following ascorbate treatment (1 mM MIA PaCa-2, 5 mM Panc-1 for 1 h) and subsequent Western blot analysis. Incubation with catalase (200 U/mL) reversed the decrease seen with ascorbate. GAPDH was used as a loading control (n = 3). Loading controls for Fig. 2C were run on a separate gel from samples derived from the same experiment. Original unprocessed blots can be found in Supplementary Fig. S2. (**D**) MMP-2 expression was decreased in 339 and PANC-1 cells following ascorbate treatment (5 mM for 1 h) and subsequent Western blot analysis. Incubation with catalase (200 U/mL) reversed the decrease seen with ascorbate. GAPDH was used as a loading control (n = 3). Original unprocessed blots can be found in Supplementary Fig. S2.

To determine the effect of P-AscH^−^ on the release of surface proteases such as matrix metalloproteinases (MMPs)^14,15^, BxPC-3 cells were treated with P-AscH^−^, resulting in decreases in invadopodia-mediated ECM degradation (Fig. 2A,B). The addition of catalase fully reverses this effect by increasing the area of degradation within the cells (Fig. 2A,B). Additionally, a 1 h treatment of P-AscH^−^ was shown to decrease the expression of MMP-9 and MMP-2 in MIA PaCa-2, PANC-1, and the patient derived cells 339 (Fig. 2C,D). Addition of catalase reverses the decrease seen in MMP-9 and MMP-2 protein expression (Fig. 2C,D). Taken together, these results show that P-AscH^−^ induced generation of H_2_O_2_ attenuates the metastatic phenotype of PDAC in vitro.

### P-AscH^−^ decreases clonogenic survival of cells in suspension without changes in viability

**Figure 3:**
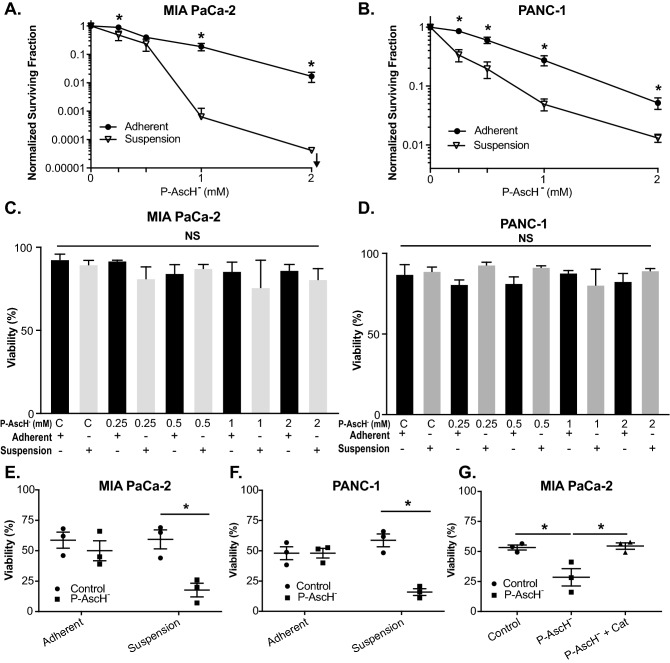
P-AscH^−^ induced susceptibility to non-adherent PDAC cells is mediated by hydrogen peroxide. PDAC cells were treated with varying doses (0–2 mM) of ascorbate for 1 h under adherent or suspension conditions. Following P-AscH^−^ treatment, cells were plated for a clonogenic cell survival assay and assessed for viability. (**A**) MIA PaCa-2 or (**B**) PANC-1 cells treated with P-AscH^−^ in suspension conditions had decreased clonogenic survival compared to PDAC cells treated in adherent conditions. Data represent normalized surviving fractions compared to controls ± SE (n = 4, *p < 0.05; Two-tailed unpaired Student’s t test comparing adherent *vs*. suspension results at each P-AscH^−^ concentration). (**C**) MIA PaCa-2 or (**D**) PANC-1 cells treated in suspension conditions with P-AscH^−^ had no changes in viability compared to PDAC cells treated in adherent conditions. Data represent percent viability ± SE (n = 4, *p* > 0.05; one-way ANOVA with Tukey’s multiple comparisons). PDAC cells were treated with ascorbate (1–2 mM) for 1 h under adherent or suspension conditions. Following P-AscH^−^ treatment, cells were subjected to fluid shear stress (FSS) and assessed for viability. (**E**) MIA PaCa-2 and (**F**) PANC-1 cells treated in suspension conditions were more susceptible to P-AscH^−^ treatment compared to PDAC cells treated in adherent conditions when assessed during FSSs. Data represent percent viability compared to controls ± SE (n = 3, *p < 0.05; two-way ANOVA with Bonferroni’s multiple comparisons). (**G**) Catalase reverses ascorbate-induced cytotoxicity during FSS. MIA PaCa-2 cells treated in suspension conditions with P-AscH^−^ were susceptible to FSS and this effect was mitigated by the addition of catalase (200 U/mL). Data represent percent viability compared to controls ± SE (n = 3, *p < 0.05; one-way ANOVA with Tukey’s multiple comparisons).

Next we wanted to investigate whether P-AscH^−^ might be effective at decreasing the survival of PDAC cells treated in suspension, which would more closely mimic the biology of CTCs. MIA PaCa-2 and PANC-1 cells were treated with varying doses (0–2 mM) of ascorbate for 1 h under adherent or suspension conditions before being plated for clonogenic cell survival assays. Both cell lines were more susceptible to P-AscH^−^ when treated under suspension conditions compared to adherent conditions (Fig. 3A,B). This effect was dose dependent and similar to previous studies of P-AscH^−^ decreasing clonogenic survival under adherent conditions^6^. However, the effect of P-AscH^−^ is more pronounced under suspension conditions, suggesting that P-AscH^−^ maybe cytotoxic to tumor cells circulating in the bloodstream of PDAC patients as well. The viability of adherent and suspension cells treated with P-AscH^−^ was tested immediately after the 1 h exposure and prior to plating for clonogenic survival assays. Trypan blue exclusion results shows that there were no differences in viability under either condition at any concentration of P-AscH^−^ (Fig. 3C,D).

### P-AscH^−^ sensitizes PDAC cells to fluid shear stress via hydrogen peroxide

To determine the effect of P-AscH^−^ on PDAC cells during exposure to hemodynamic forces including fluid shear stress (FSS), cells were treated with P-AscH^−^ under adherent and suspension conditions and then exposed to FSS as described previously^16^. Both MIA PaCa-2 and Panc-1 cells treated in suspension conditions were more susceptible to FSS compared to cells treated under adherent conditions (Fig. 3E,F). Importantly, cells remained viable with P-AscH^−^ while in suspension (Fig. 3C,D) but there were differences in viability during exposure to FSS (Fig. 3E,F), as we have shown that dead/dying cells are mechanically fragile and highly susceptible to destruction by FSS^11^. Interestingly, the basal levels of FSS resistance we observed in both of these PDAC cell lines is lower than that observed for many other cell lines^16^, which necessitated using lower levels of FSS in these experiments (flow rate of 200 μL/s vs. 250 μL/s). The addition of catalase reverses the sensitization to FSS by P-AscH^−^ in MIA PaCa-2 cells (Fig. 3G). Catalase reversal of sensitization to FSS by P-AscH^−^ was also seen in the PANC-1 cell line where control treatment decreased viability to 47%, which was further reduced to 36% with P-AscH^−^ (2 mM), but reverses to 50% with catalase and P-AscH^−^ treatment. These data further suggest that the catalase reversal of sensitization to FSS by P-AscH^−^ is mediated by hydrogen peroxide.

### P-AscH^−^ decreases the metastatic phenotype of PDAC in vivo

**Figure 4:**
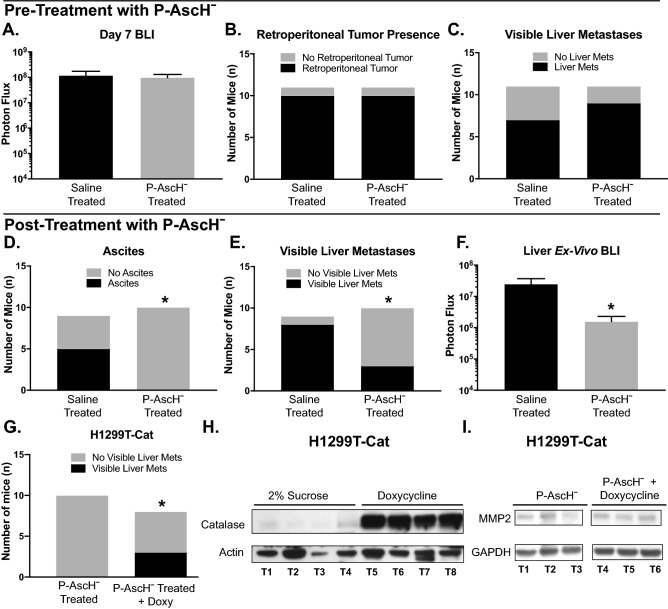
P-AscH^−^ decreases the metastatic potential of PDAC in vivo**.** MIA PaCa-2-Luc-GFP or H1299T-Cat (2 × 10^6^) cells were injected into the spleen and a splenectomy was performed. One set of mice were pre-treated with I.P. P-AscH^−^ (4 g/kg) or saline (1 M) twice a day for two days prior to splenic injection, the other set of mice were treated with P-AscH^−^ or saline twice a day starting 2 days following splenic injection. Tumor formation was followed for a total of 30 days. (**A**) Bioluminescence imaging 7 days following tumor cell injection showed no difference in photon flux between saline treated mice and mice treated with P-AscH^−^. Data represent the mean photon flux compared to controls ± SE (n = 5, *p* > 0.05; Two-tailed unpaired Student’s t test). (**B**) P-AscH^−^ treated mice were found to have a no statistically different chance of forming retroperitoneal tumors compared to saline treated mice. Data are represented in a Two-tailed contingency table (n = 11, *p* > 0.05; Fisher’s exact test). (**C**) P-AscH^−^ treated mice were found to have a no statistically different chance of forming visible liver metastases compared to saline treated mice. Data are represented in a Two-tailed contingency table (n = 11, *p* > 0.05; Fisher’s exact test). (**D**) Saline treated mice were found to have a greater incidence of developing ascites compared to P-AscH^−^ treated mice. Data are represented in a Two-tailed contingency table (n = 9–10, *p < 0.05; Fisher’s exact test). (**E**) Saline treated mice were found to have a statistically greater chance of visible liver metastases compared to P-AscH^−^ treated mice. Data are represented in a Two-tailed contingency table (n = 9–10, *p < 0.05; Fisher’s exact test). (**F**) A significant decrease was seen in *ex-vivo* bioluminescence of livers in saline treated mice compared to P-AscH^−^ treated mice. Data represent the mean photon flux compared to controls ± SE (n = 9–10, *p < 0.05; Two-tailed unpaired Student’s t-test). (**G**) Mice injected with H1299T-CAT cells were treated with control (2% sucrose) or doxycycline (2 mg/mL and 2% sucrose) in drinking water (changed every 2–3 d). Data are represented in a Two-tailed contingency table (n = 8–10, *p < 0.05; Chi-square test). (**H**) Catalase immunoreactive protein is increased in tumors from mice treated with P-AscH^−^ + doxycycline in their drinking water compared to mice treated with P-AscH^−^. Tumors were excised and western blotting was performed. Actin was used as a loading control. (Representative blots shown, n = 3). Original unprocessed blots can be found in Supplementary Fig. S3. (**I**) MMP-2 immunoreactive protein is decreased in mice treated with P-AscH^−^ compared to mice treated with P-AscH^−^ + doxycycline in their drinking water. Tumors were excised and western blotting was performed. GAPDH was used as a loading control. (Representative blots shown, n = 3). Original unprocessed blots can be found in Supplementary Fig. S3.

First, we sought to determine if P-AscH^−^ treatment impacted the microenvironment of metastatic end organs to inhibit metastasis formation by splenic injection of PDAC cells expressing both luciferase and GFP. Results indicated that treatment with P-AscH^−^ prior to splenic injection had no effect on initial bioluminescence, retroperitoneal tumor formation, or visible liver metastases compared to saline treated mice (Fig. 4A–C). In comparison, P-AscH^−^ after splenic injection, decreases ascites development, visible liver metastases, and *ex-vivo* liver bioluminescence after 30 days compared to saline treated mice (Fig. 4D–F). To demonstrate that the effect of P-AscH^−^ treatment was due to the generation of hydrogen peroxide, doxycycline inducible catalase expressing H1299T cells were injected into the spleens of mice. Mice treated with P-AscH^−^ and doxycycline were found to have visible liver metastases while mice treated with P-AscH^−^ alone did not (Fig. 4G). Catalase expression was induced in mice treated with doxycycline (Fig. 4H). In addition, mice treated with P-AscH^−^ alone show decreases in MMP-2 expression compared to mice treated with P-AscH^−^ and doxycycline (Fig. 4I), consistent with the in vitro studies in Fig. 2D.

**Figure 5:**
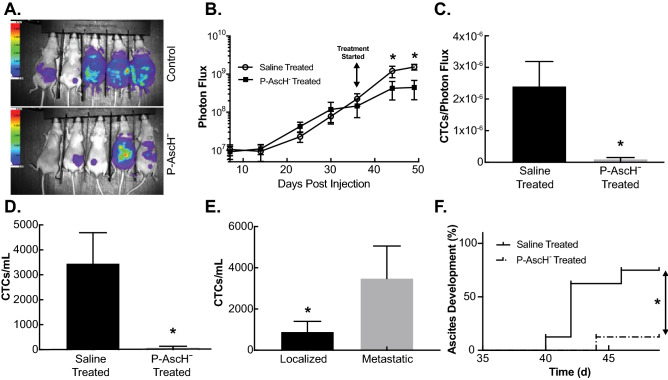
P-AscH^−^ slows PDAC tumor growth, reduces ascites development and decreases CTCs in vivo. (**A**) The growth of the pancreatic tumors was tracked weekly using bioluminescent imaging. Pancreatic tumors were formed through ultrasound guided orthotopic injections of pancreatic tumors cells into the pancreas of nude mice. The representative images show tumor burden between saline treated mice and P-AscH^−^ treated mice on day 49 of the experiment. (**B**) P-AscH^−^ significantly decreased tumor growth over time as determined by bioluminescent imaging. Treatments of P-AscH^−^ or saline were started on day 35 following tumor cell injections. A region of interest was placed around each mouse image and total photon flux (photons per second) was quantified A significant decrease in tumor growth rate was seen after initiation of therapy as indicated by bioluminescent signal strength. Data represent the mean photon flux compared to controls ± SE (n = 6–10, *p < 0.05; one-way ANOVA with Bonferroni’s multiple comparisons). (**C**) Saline treated mice were found to have significantly increased CTCs per tumor burden as measured by photon flux compared to P-AscH^−^ treated mice (2.4 × 10^–6^ CTCs/photon flux *vs* 9.3 × 10^–8^ CTCs/photon flux). Data represent the mean of CTCs/photon flux ± SE (n = 6–10, *p < 0.05; Mann–Whitney). (**D**) P-AscH^−^ decreased the number of CTCs. Saline treated mice were found to have increased CTCs/mL compared to P-AscH^−^ treated mice (3,460 +/− 1232 CTCs/mL *vs* 80 +/− 50 CTCs/mL) Data represent the mean of CTCs/mL ± SE (n = 6–10, *p < 0.05; Mann–Whitney). (**E**) The number of CTCs was increased in mice with metastatic disease. CTCs were isolated on day 50 of the experiment following a cardiac blood draw. Mice with localized tumors were found to have fewer CTCs/mL than mice with evidence of metastatic disease (900 +/− 505 CTCs/mL *vs* 3,590 +/− 1570 CTCs/mL). Data represent the mean of CTCs/mL ± SE (n = 8, *p < 0.05; Mann–Whitney). (**F**) Mice were evaluated daily for ascites accumulation following pancreatic injection of tumor cells. P-AscH^−^ decreased the percentage of mice with ascites (n = 8, *p < 0.05) compared to control (log-rank)**.**

Finally, luciferase and GFP expressing MIA PaCa-2 cells were injected directly into the pancreas through ultrasound guidance. Tumors formed for 35 days prior to treatment initiation in order to permit metastases to colonize other sites. On day 35, mice were randomized based on whole body BLI into two groups with similar mean BLI prior to receiving daily P-AscH^−^ or saline. On day 50, all mice were imaged for tumor burden assessment and blood was collected for CTC analysis. P-AscH^−^ treatment decreases tumor growth over time compared to control mice (Fig. 5A,B). P-AscH^−^ treatment decreases the absolute number of CTCs (80 CTCs/mL ± 50) compared to saline treated mice (3460 CTCs/mL ± 1232) (Fig. 5C). This difference was still observed even when the CTC numbers were normalized to the tumor burden, as determined by BLI (Fig. 5D). Also, mice with metastatic disease had increases in the number of CTCs when compared to mice that only had localized disease (Fig. 5E). Additionally, comparisons between groups show that treatment with P-AscH^−^ delays the formation of ascites (Fig. 5F). Interestingly, tumor cells isolated and cultured from visible metastatic hepatic lesions in both groups of mice as well as the parental cell line initially injected show no differences in clonogenic survival after being re-challenged with P-AscH^−^ (Supplemental Figure 4). Taken together, these results demonstrate that P-AscH^−^ decreases the metastatic potential of PDAC in vivo and that the effects are mediated by a peroxide-dependent mechanism.

### P-AscH^−^ decreases nuclease activity in patients with metastatic PDAC

**Figure 6:**
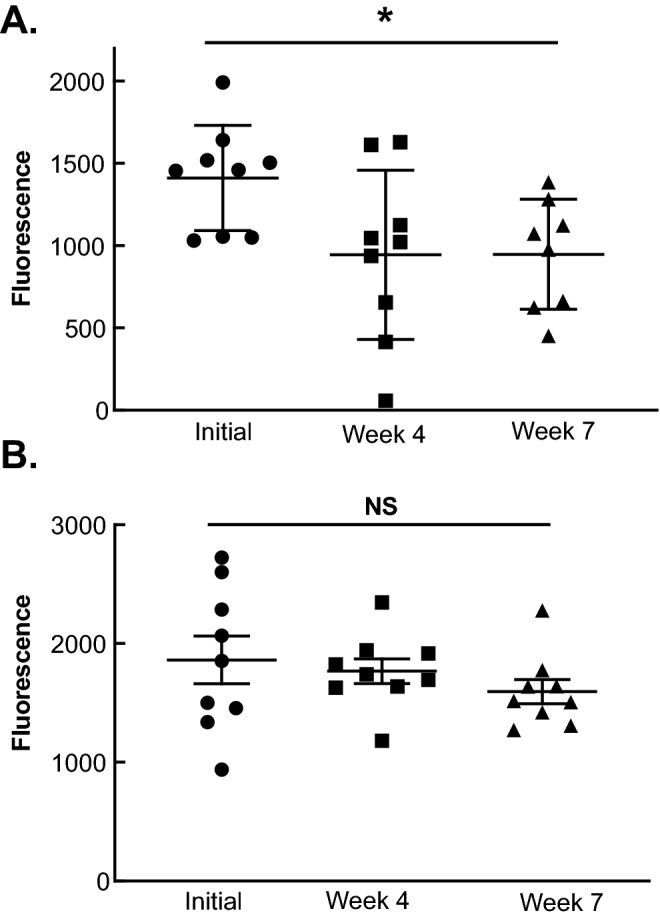
P-AscH^−^ treatment attenuates nuclease activity in patients with metastatic PDAC. Subjects with metastatic PDAC treated with P-AscH^−^ had decreases in nuclease activity compared to subjects with local and regional disease treated with P-AscH^−^. (**A**) Subjects in the University of Iowa clinical trial (NCT 01049880) treated with P-AscH^−^ and gemcitabine had decreased nuclease activity over time. Initial nuclease activity was compared to nuclease activity at weeks 4 and 7 of treatment. Data represent the mean nuclease activity compared to control ± SE (n = 9, *p < 0.05; unpaired Students T-test). (**B**) Subjects in the University of Iowa clinical trial (NCT 01852890) treated with P-AscH^−^, gemcitabine, and radiation showed minimal differences in nuclease activity. Initial activity was compared to activity at weeks 4 and 7 of treatment (n = 9, *p* > 0.05; unpaired Students T-test).

To evaluate the effect P-AscH^−^ might have on CTC nucleases, we compared plasma samples from subjects with metastatic PDAC receiving concurrent gemcitabine and P-AscH^−^ (NCT 01049880)^7^ and in subjects with local–regional disease where P-AscH^−^ was infused during radiation therapy and receiving concurrent gemcitabine (NCT 01852890)^6^. Plasma tested from patients with metastatic disease show a significant decrease in nuclease activity over the course of treatment while patients presenting with local and regional disease did not (Fig. 6A,B). These results support the in vivo studies demonstrating that P-AscH^−^ reduces metastatic colonization and decreases the survival of CTCs in advanced PDAC.

## Discussion

P-AscH^−^ is a prototypical antioxidant/pro-oxidant that can elicit a protective response in normal cells but is toxic to tumor cells^4,17–20^. Our in vitro, in vivo^4,6,21^, and human studies^6,7^ show that P-AscH^−^ mediated cell death is due to the generation of H_2_O_2_ via ascorbate radical formation, with ascorbate as the electron donor. Several phase I clinical trials of P-AscH^−^ in patients with advanced cancers have been performed. The focus of these initial trails was on pharmacokinetics, dosing, resulting ascorbate plasma concentrations, and safety. They demonstrate that intravenous administration of P-AscH^−^ can achieve plasma concentrations as high as 1–30 mM and show that P-AscH^−^ is safe, well tolerated and with potential efficacy^6,7,22^. Phase I trials (NCT 01049880 & NCT 01852890)^6,7^ from our group demonstrated an increase in progression free survival, suggesting that P-AscH^−^ may reduce the metastatic disease burden in PDAC. Our current study demonstrates that P-AscH^−^ inhibits the mechanisms involved in metastatic disease in PDAC. Additionally, our animal models show that P-AscH^−^ reduces the capacity for PDAC cells to exist in the circulation.

A hallmark component of cancer cell metastasis is the ability for cells to migrate and invade through the extracellular matrix. In PANC-1 PDAC cells, Polireddy et al. demonstrated a decrease in migration and invasion^22^. Similarly, this result was seen in gastric cancer, and the effects were reversed with the addition of catalase^23^. Our current study corroborates their findings and extends these observations by demonstrating that P-AscH^−^ generation of H_2_O_2_ mediates this decrease in invasion. One of the first steps of tumor cell invasion is the release of surface proteases such as matrix metalloproteinases (MMPS)^14,15^. MMPS are secreted by invadopodia, which are actin-rich structures present a the basal surface of cancer cells (typically with high metastatic potential) that can cross extracellular barriers and degrade the ECM^24,25^. Previously, P-AscH^−^ decreased MMP-2 expression^22^ which is required for the assembly of functional invadopodia in invasive cancer cells^26^. Our current study expands these findings by again demonstrating P-AscH^−^ decreases in MMP-2 expression and invadopodia-mediated ECM degradation via a hydrogen peroxide mediated mechanism.

The systemic response of PDAC to treatment is difficult to objectively assess. Currently, clinicians rely on changes in tumor size and location on conventional imaging to determine the extent of disease progression^27^. Circulating tumor cells (CTCs), however, do offer insight into systemic disease behavior and have been used in PDAC and other malignancies to predict tumor progression and measure response to treatment^28–32^. CTCs represent an intermediate in metastatic colonization because they first need to survive detachment induced cell death (anoikis). Although P-AscH^−^ significantly impairs clonogenic survival in several PDAC cell lines^4^, these assays have been previously done by treating cells under adherent conditions. Since PDAC cells have been shown to form metastases by entering the blood stream^3^, we hypothesized that P-AscH^−^ might be effective at decreasing the survival of PDAC cells treated in suspension, which would more closely mimic the biology of circulating tumor cells. Our current study shows that P-AscH^−^ had a more potent effect on PDAC cells when they were treated in suspension rather than under adherent conditions. Although this treatment did not affect short-term viability, it did significantly reduce the clonogenic potential of these cells. This result is consistent with studies demonstrating that detachment-induced cell death results from increased oxidative burden^33^.

While in circulation, CTCs are exposed to a fluid microenvironment in the bloodstream that is quite different from the environment cancer cells typically encounter in solid tissues. One aspect of this microenvironment is exposure to hemodynamic forces including fluid shear stress^34^. Studies have shown that cells exposed to FSS are in a more oxidative state^35^ and that oxidative stress can inhibit the formation of metastasis^9^. In this study, P-AscH^−^ sensitized PDAC cells in suspension to FSS-induced cell death. Moreover, the effect was shown to be mediated by hydrogen peroxide when the addition of catalase reversed the effect. Our previous work has demonstrated that transformed cells from many histologic origins, including PDAC cells, are resistant to FSS as compared to non-transformed controls^16^. FSS resistance is, in part, driven by RhoA-actomyosin dependent mechano-adaptation of CTCs to FSS^11^. Exposure to FSS has been shown to induce oxidative stress^36^, suggesting that P-AscH^−^ treatment further advances PDAC to a more oxidative environment leading to toxicity.

The experimental studies that support PDAC progression in which the seeding of distant organs occurs before, and in parallel to, tumor formation at the primary site^3^ are consistent with the clinical nature of PDAC as the majority of patients have metastatic disease at the time of diagnosis. Greater than 75% of patients who undergo surgical resection for non-metastatic PDAC and have negative surgical margins, still die from metastatic disease within 5 years^37^. Our current study shows that P-AscH^−^ can decrease the metastatic potential of PDAC in vivo. In a primary tumor model, P-AscH^−^ slows tumor growth, reduces visible hepatic metastases and ex-vivo liver bioluminescence, reduces ascites formation, and decreases levels of CTCs. However, P-AscH^−^ prior to splenic injection of PDAC cells did not decrease bioluminescence, retroperitoneal tumor formation, or visible liver metastases compared to control mice, suggesting that P-AscH^−^ does not alter the environment to impede metastatic colonization but instead exerts its effects directly on the metastatic PDAC cells.

Previous studies have demonstrated that oxidative stress inhibits distant tumor metastases in an in vivo model^9^. Successful melanoma metastases underwent reversible metabolic changes during metastasis that increased their capacity to withstand oxidative stress. Antioxidants promoted distant metastasis in tumor bearing mice without significantly affecting the growth of subcutaneous tumors. In addition, detachment-induced reactive oxygen species has been found to inhibit fatty acid oxidation during ATP deficiency that occurs following detachment of epithelial cells from the ECM^33^. Antioxidants can reverse this inhibition and assist in survival and enhance colony formation^33^. Similarly, our in vivo findings utilizing H1299T-CAT cells expressing doxycycline inducible catalase, show that the downregulating effects of P-AscH^−^ on MMP-2 expression can be reversed when the antioxidant catalase is present. Combined, these findings suggest that oxidative stress may limit distant metastasis, which supports our hypothesis of using P-AscH^−^ to induce oxidative stress, leading to decreases in metastatic disease in PDAC.

Cell-free circulating tumor DNA (ctDNA) has shown promise as a biomarker to improve early tumor detection, prognostic stratification, and monitoring of tumor dynamics. First, detection of mutant KRAS ctDNA prior to surgery or in the immediate postoperative period predicts a higher likelihood of tumor recurrence and poorer survival^38^. Second, rising mutant KRAS ctDNA during postoperative follow-up anticipates radiographic/clinical recurrence^38^. Current studies have demonstrated potential clinical applicability in longitudinal monitoring of PDAC subjects to provide predictive and prognostic information that was relevant to therapeutic decisions^39^, while others have shown that CTC-derived nucleases can be exploited for signal amplification in detection methods^40^. P-AscH^−^ decreases CTC-derived nucleases in subjects with stage 4 metastatic PDAC while P-AscH^−^ treatment had little effect on CTC-derived nucleases in patients with loco-regional PDAC without distant metastases. Taken together, these data suggest that P-AscH^−^ may be beneficial in decreasing metastatic disease.

In summary, P-AscH^−^ inhibits invasion and degradation of the basement membrane as well as MMPs in PDAC. Through a peroxide mediated mechanism, P-AscH^−^ decreases clonogenic survival along with viability during fluid shear stress of PDAC cells in suspension, while decreasing CTCs, hepatic metastases, and development of ascites in different models of PDAC metastases. In human studies, P-AscH^−^ decreases circulating tumor cell-derived nucleases in subjects with stage IV PDAC. Further studies have been initiated by our group to determine the clinical utility of P-AscH^−^ in a current, ongoing randomized phase II trial (www.clinicaltrials.gov, NCT 02905578).

## Methods

### Cell culture and reagents

Human PDAC cell lines MIA PaCa-2 and PANC-1 were cultured in DMEM (Gibco, 11965) supplemented with 10% FBS (Gibco, 26140) and 1% penicillin–streptomycin antibiotic (Gibco, 15140) while BxPC-3 was cultured in RPMI (Gibco, 11875) with 10% FBS and 1% penicillin–streptomycin antibiotic. The patient derived cell line 339 was obtained from the Medical College of Wisconsin surgical oncology tissue bank and cultured in Dulbecco’s Modified Eagle’s Media Nutrient Mixture F-12 (Gibco, 11320) supplemented with 5% FBS, penicillin/streptomycin, human recombinant EGF (Gibco, PHG0311), bovine pituitary extract (Gibco, 13028), hydrocortisone (Sigma, H0888), and human recombinant insulin (Gibco, 12585) according to instructions^41,42^. The human non-small cell lung carcinoma cell line overexpressing catalase, H1299T-CAT, was generated as previously described^43,44^ and cultured in RPMI supplemented with 10% FBS. Luciferase and GFP expressing MIA PaCa-2 cells were generated using pQClucIN and pMSCV-IRES-GFP plasmids as previously described^45,46^.

Human pancreatic ductal adenocarcinoma cell lines MIA PaCa-2, PANC-1, and BxPC-3 were purchased directly from American Type Culture Collection and no additional authentication was performed. All cells were passaged routinely for less than 6 months and not used above passage 20. The H1299T cells were characterized and verified by IDEXX-RADIL. Mycoplasma testing was performed routinely on cells every 6 months. Regardless of varying cell type and media components, all cells were treated in fresh 10% DMEM media with ascorbate for 1 h at 37 °C. Ascorbate came from a stock solution of 1 M (pH 7) made under argon and stored with a tight-fitting stopper at 4 °C. Ascorbate concentration was checked at 265 nm, ε = 14,500 (mol/L)^−1^ cm^−1^. To account for variation in media concentration and cellular metabolism among cell lines, final concentrations were calculated in units of moles-per-cell as described^47,48^.

### Invasion assay

PDAC cell lines 339, BxPC-3, and PANC-1 in 60 mm dishes were treated with P-AscH^−^ (2–4 mM) with or without catalase (200 U/mL) for 1 h. Following treatment, 1–3 × 10^5^ cells in 200 µL DMEM were seeded into Matrigel inserts (8-μm pore size) (Corning, 354,483) and incubated for 24 (PANC-1) to 48 h (BxPC3 and 339) at 37 °C in DMEM containing 10% FBS. Non-invading cells from the upper membrane surface were removed by a cotton tip swab. Invading cells were fixed by ice-cold methanol and stained with Giemsa and the number of invading cells counted from five random fields.

### ECM degradation assay

The ECM degradation assay was performed as described by Artym et al.^49^ Briefly, glass coverslips (18 mm) were washed in 20% nitric acid and coated with poly-l-lysine (50 µg/mL). The coverslips were then coated with Oregon Green-conjugated gelatin (Thermo Fisher Scientific, G13186). BxPC-3 cells were treated with 1 mM ascorbate with or without catalase (200 U/mL) for 1 h. Following treatment, cells (5 × 10^4^) in 1 mL 10% RPMI were re-plated on coverslips covered with Oregon Green-conjugated gelatin in a 12-well plate for 24 h. Cells were fixed with 2% paraformaldehyde, permeabilized with 0.5% Triton X-100, and stained for actin using TRIC-Phalloidin. Fluorescence images were acquired with a Zeiss 510 at 63×/1.4 N.A. objective. Foci of degraded matrix were visible as black spots in the bright fluorescent gelatin matrix.

### Western blot analysis

Cells or tumors were lysed in RIPA buffer and pelleted by centrifugation. Protein concentrations were determined using a Bio-Rad DC Bradford Protein Assay (Bio-Rad Laboratories). 40 μg of protein was electrophoresed in a Bio-Rad 4–20% Precast Gel for 65 min at 120 V. The proteins were electro-transferred onto a PVDF membrane, and blocked with 5% nonfat milk in 0.1%Tween-PBS (TPBS) for 60 min. The membranes were incubated with MMP-2 and MMP-9 (1:1000, Cell Signaling Technology, 40994S and 2270S) or Catalase (1:1000, Cell Signaling Technology, 14097S) at 4 °C overnight. Membranes were washed 5 times with TPBS and incubated with secondary antibodies conjugated with horseradish peroxidase (1:50 000, Millipore,). GAPDH (1:5000, Millipore, MAB374) or Actin (1:1000, Cell Signaling Technology, 4970) was used as a loading control. After wash with TPBS, membranes were stained with Super Signal West Pico Chemiluminescent Substrate (Thermo Fisher Scientific, 34580) and exposed to Classic Blue Autoradiography Film.

### Clonogenic cell survival

MIA PaCa-2 and PANC-1 cells were treated with varying doses (0–2 mM) of P-AscH^−^ for 1 h under adherent or suspension conditions before being plated for a clonogenic survival assay. Clonogenic survival assays were performed as previously described^6^. Briefly, treated cells were counted and plated into 6 well tissue culture plates at 500–3000 cells/well. Cells formed colonies for 7–14 days before being fixed and stained for analysis. Colonies containing ≥ 50 cells were scored.

### Viability assay

Cells were trypsinized with TrypLE Express (Gibco, 12604) to form a single cell suspension and combined with equal parts 0.4% Trypan Blue stain. Cell viability was then determined on a Countess II automated cell counter (Thermo Fisher Scientific) according to manufacturer instructions. Data is reported as percent viability.

### Fluid shear stress

Fluid shear stress assays were performed as previously described^16^. Briefly**,** MIA PaCa-2 and PANC-1 cells were suspended in DMEM without serum at a concentration of 5 × 10^5^ cells/mL. The suspension was placed in a polypropylene tube and a sample taken for a static control. The samples were repeatedly pulsed through the needle at 200 µL·s^−1^ 10 times using a syringe pump (Harvard Apparatus, PDH-2000). A sample was taken after the 10th exposure, and the concentration of trypan blue excluding cells is measured for the static control and sheared cells. Viability was determined by dividing the concentration of cells that had been sheared by the concentration of cell held under static condition.

### In vivo tumor implantation

All animal protocols were reviewed and approved by the Animal Care and Use Committee of The University of Iowa. All experiments were performed in accordance with the approved guidelines and regulations. Female six-week-old athymic-nu/nu mice (*Foxn*1^nu^) were purchased from Envigo and allowed to acclimate in the unit for 1 week before any manipulations were performed. In the first series of in vivo experiments, MIA PaCa-2 cells expressing luciferase and GFP or H1299T-CAT cells (2 × 10^6^) were injected into the spleens of nude mice (10–11 per group) and a splenectomy was performed 1 min following tumor cell injection^50^. The abdomen was closed with absorbable suture and skin clips. One set of mice (MIA PaCa-2 only) were treated with I.P. P-AscH^−^ (4 g/kg) or saline (1 M) B.I.D. for two days prior to splenic injection, while the other set of mice were treated with P-AscH^−^ or saline B.I.D. starting 2 days following tumor cell injection. Mice injected with H1299T-CAT cells were treated with control (2% sucrose) or doxycycline (2 mg/mL and 2% sucrose) in drinking water (changed every 2–3 d). For mice injected with MIA PaCa-2luc cells, tumor formation and growth were monitored weekly for 30 d through bioluminescent imaging immediately after I.P. luciferin injection (200 µL of a 15 mg/mL solution of VivoGlo luciferin) (Promega, P104) on an Ami HTX imager (Spectral Instruments Imaging). Total photon flux (photons per second) was quantified (AMIView software) by placing and area of interest around each mouse. The animals were euthanized on day 30, and necropsy was performed to determine the presence or absence of ascites, the formation of a retroperitoneal tumor, and the presence of visible liver metastases prior to euthanasia. Ex vivo imaging was performed as previously described^46^. Briefly, mouse livers were removed postmortem and ex vivo imaging was performed to confirm the presence of hepatic metastatic disease. Bioluminescence was used to identify the presence of luciferase expression. In the second series of in vivo experiments, orthotopic intrapancreatic injections and bioluminescent imaging was performed as previously described^46,51^. MIA PaCa-2 cells (4 × 10^5^) expressing luciferase and GFP were suspended in a 1:1 mixture of PBS and Matrigel and injected orthotopically (10 mice per group) under ultrasound guidance. Tumor formation and growth were monitored weekly through bioluminescent imaging as stated above. Tumor progression was followed for 50 days post cancer cell injection. Mice were treated with P-AscH^−^ (twice daily I.P. injection, 4 g/kg) or saline (1 M) on day 35 post tumor cell injections.

### Circulating tumor cell (CTC) isolation and analysis

CTC isolation and analyses were performed as previously described^46^. Cardiac blood draws were performed on day 50 of the experiment using heparinized needles and syringes to obtain roughly 1 mL of circulatory volume. Blood samples underwent immediate red blood cell lysis and the resulting supernatant was aspirated and the procedure was repeated. Red blood cells were resuspended in FACs buffer that contained 1 × 10^5^ polystyrene FluoSpheres (Molecular Probes, F8843, 15 µm scarlet) per mL. CTCs in each sample were detected and quantified using flow cytometry and a known concentration of microspheres. Samples were run on an LSR II (BD Biosciences, λ_ex_ = 645 nm and λ_em_ = 680 nm) flow cytometer and analysis performed utilizing FlowJO software (BD Biosciences). Results were used to determine circulating tumor cell concentration (CTC/mL) for each mouse by the equation [number of CTCs detected/ (FluoSpheres detected/100,000 FluoSpheres per mL)].

#### Hepatic tumor cell isolation

Hepatic tumor cell isolation was performed as previously described^46^. Liver specimens with visible metastatic lesions were used to isolate and grow single tumor cells from mice. Hepatic sections were quickly minced with a scalpel in cold HBSS and centrifuged before the addition of 0.5% w/v collagenase (Gibco, 17100). Cells were plated and grown for further analysis.

#### Nuclease activity assay

The CTC-derived nuclease activity assay was performed as previously described^40^. Plasma samples were collected from subjects with stage 4 metastatic pancreatic cancer enrolled in a phase I trial where P-AscH^−^ was combined with gemcitabine^7^ approved by The University of Iowa Human Institutional Review Board and the Protocol Review and Monitoring Committee of the Holden Comprehensive Cancer Center at The University of Iowa Hospitals and Clinics on May 22, 2008. Informed consent was obtained from all participants and all research was performed in accordance with the approved guidelines and regulations. The trial was listed on www.clinicaltrials.gov under NCT 01049880. Nuclease activity was also determined in a separate phase I trial involving local–regional pancreatic cancer without distant metastases^6^. This phase I trial was approved by The University of Iowa Human Institutional Review Board and the Protocol Review and Monitoring Committee of the Holden Comprehensive Cancer Center at The University of Iowa Hospitals and Clinics on December 30, 2014 and listed on www.clinicaltrials.gov under NCT 01852890. Informed consent for both studies were documented by use of a written consent form approved by the Investigational Review Board and The University of Iowa. Nuclease-activated probes were combined with 10 µL of plasma and incubated for 6 h at 37 °C prior to being measured for fluorescence intensity (λ_ex_ = 485 nM and λ_em_ = 528 nM).

#### Statistical methods

Data are presented as the mean ± SEM. For statistical analyses of two groups, unpaired two-tailed Student’s *t*-test were utilized. For statistical analyses of two nonparametric groups, Mann–Whitney tests were used. To study statistical differences between multiple comparisons, significance was determined using either a one-way ANOVA analysis or a two-way ANOVA analysis with Tukey’s or Bonferroni’s multiple-comparisons tests as stated in the figure legends. To determine if non-random associations between two categorical variables existed, a Fisher’s exact test was used. To compare statistical analysis of survival distributions, log-rank tests were utilized. All analyses were performed in GraphPad Prism (GraphPad Software, Inc.).

## Supplementary information

Supplementary Figures.

## Data Availability

No datasets were generated or analyzed during the current study.
