# Supplementary material for: Pharmacological ascorbate inhibits pancreatic cancer metastases via a peroxide-mediated mechanism
Source: Sci Rep. 2020 Oct 19;10:17649. doi: 10.1038/s41598-020-74806-2 (PMC7572461; doi:10.1038/s41598-020-74806-2)
Supplement: Supplementary file 1 — Supplementary Figures. [file 41598_2020_74806_MOESM1_ESM.pdf]

***SUPPLEMENTARY MATERIALS FOR:***

# PHARMACOLOGICAL ASCORBATE INHIBITS PANCREATIC CANCER METASTASES VIA A PEROXIDE-MEDIATED MECHANISM

Brianne R. O'Leary[1]
Matthew S. Alexander[1]
Juan Du[1]
Devon L. Moose[2]
Michael D. Henry[2,3,4,5,6]
Joseph J. Cullen[1,3,6]

Department of Surgery[1], Department of Molecular Physiology and Biophsics[2], The Holden Comprehensive Cancer Center, University of Iowa Hospitals and Clinics[3], Department of Pathology[4], Department of Urology[5], Free Radical and Radiation Biology Program, Department of Radiation Oncology[6], The University of Iowa Carver College of Medicine, Iowa City, Iowa, USA.

* Correspondence Author

Joseph J. Cullen, M.D.,

1528 JCP, 200 Hawkins Drive, University of Iowa Hospitals and Clinics, Iowa City, IA 52242.

joseph-cullen@uiowa.edu W: (319) 353-8297, Fax: (319) 356-8378.

**A**

**Control** **P-AscH$^-$** **P-AscH$^-$ + Cat**

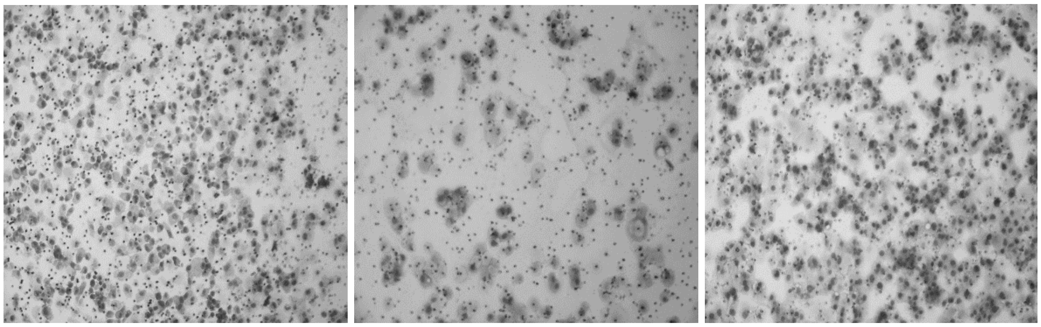

**B**

**Control** **P-AscH$^-$** **P-AscH$^-$ + Cat**

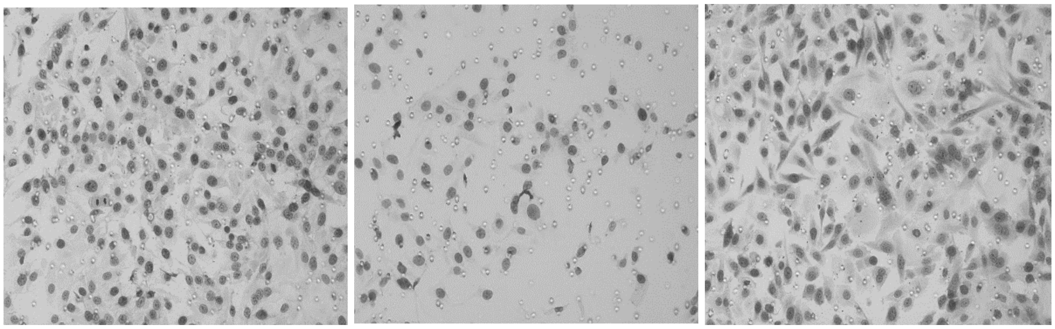

**Supplemental Figure 1. P-AscH$^-$ attenuates the invasive phenotype of PDAC *in vitro.***

**A & B.** Representative invasion images from Panc-1 (A) and 339 (B) PDAC cells in the presence of P-AscH$^-$ (4mM) and/or catalase (200 U/mL). Cells were treated for 1 h then seeded at 1-3 x $10^5$ and incubated for 24 (PANC-1) or 48 h (339).

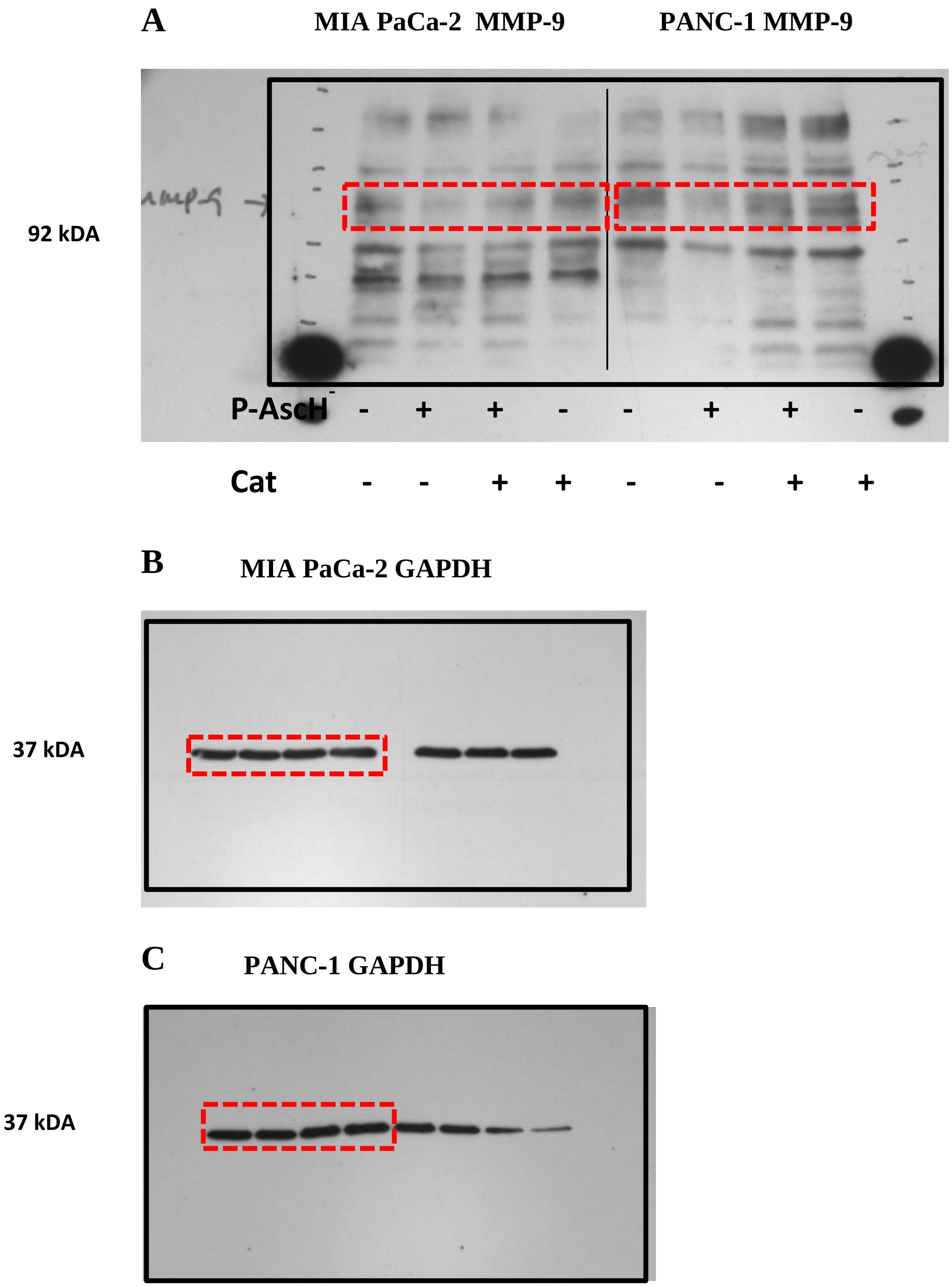


**Supplementary Figure 2.** Original unprocessed Western blots from main Figure 2C. **A.** MMP-9 expression from MIA PaCa-2 and PANC-1 cells following treatment with ascorbate +/- catalase. **B.** Corresponding GAPDH loading control from MIA PaCa-2 cells. **C.** Corresponding GAPDH loading control from PANC-1 cells.

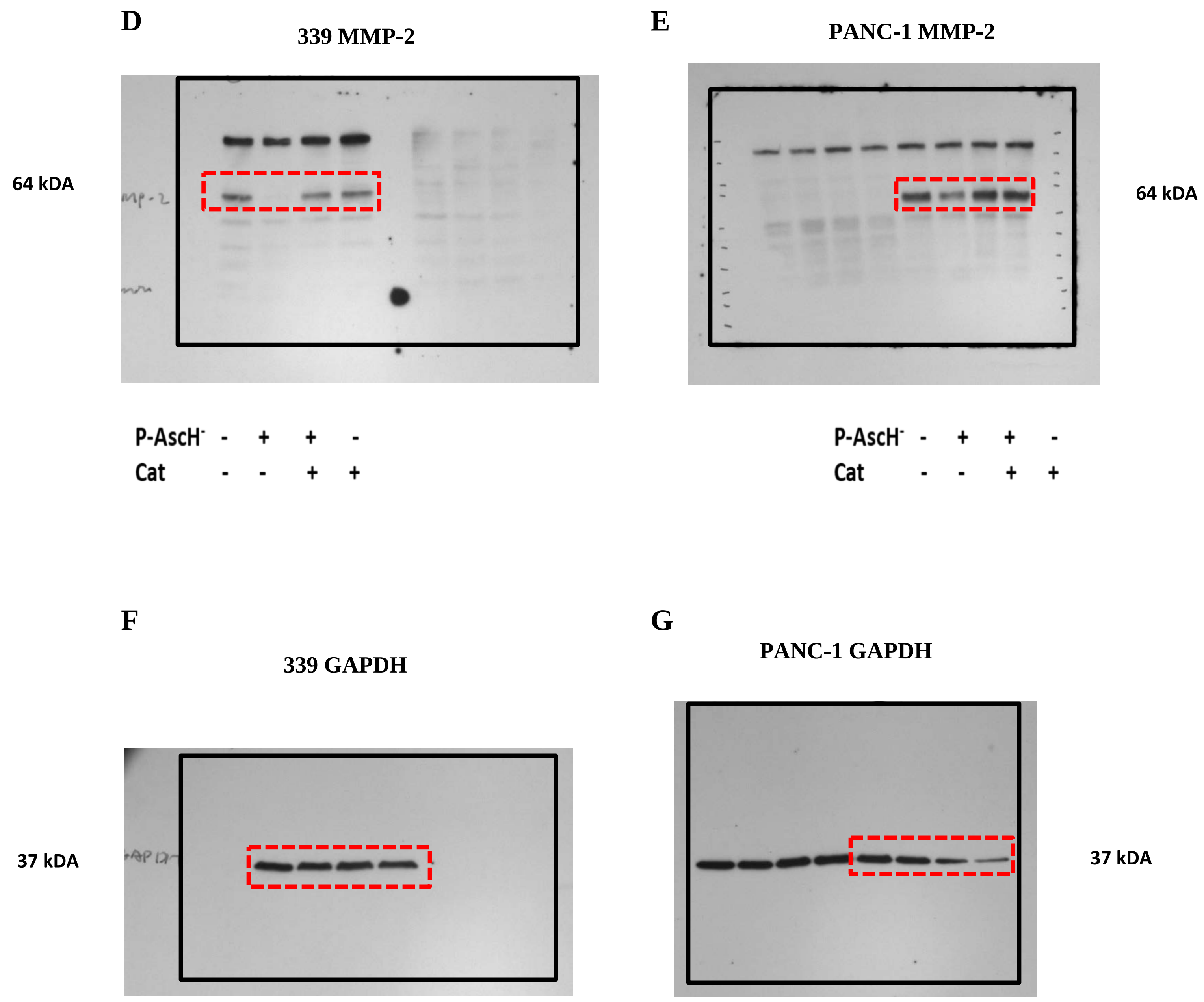


**Supplementary Figure 2 Continued.** Original unprocessed Western blots from main Figure 2D. **D & E.** MMP-2 expression from 339 and PANC-1 cells following treatment with ascorbate +/- catalase. **F.** Corresponding GAPDH loading control from 339 cells. **G.** Corresponding GAPDH loading control from PANC-1 cells.

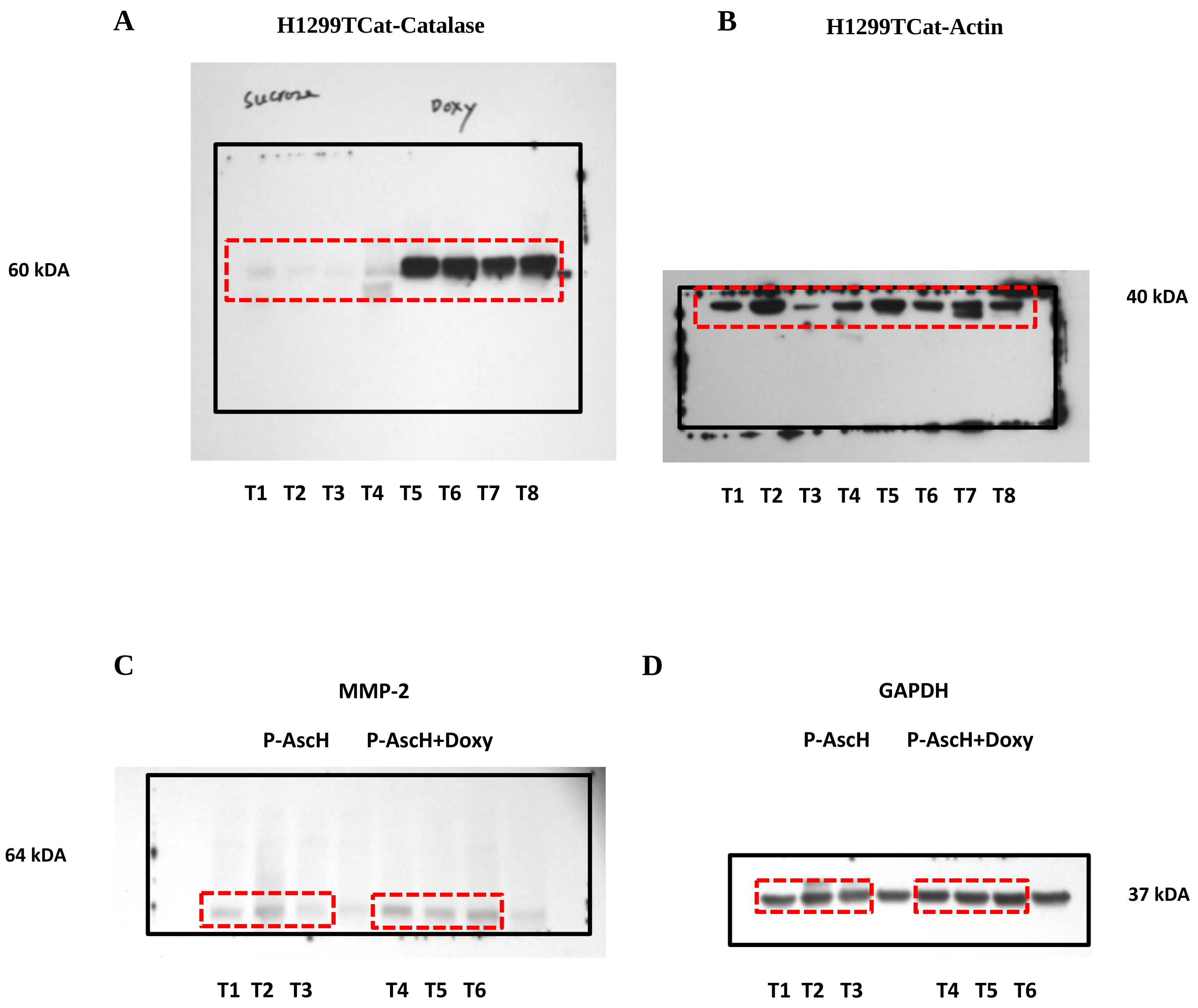


**Supplementary Figure 3.** Original unprocessed Western blots from main Figures 4H&I. **A.** Catalase immunoreactive protein is increased in tumors from mice treated with P-AscH$^-$ + doxycycline. **B.** Corresponding Actin loading control from mouse tumors. **C.** MMP-2 immunoreactive protein is decreased in mice treated with P-AscH$^-$ compared to mice treated with P-AscH$^-$ + doxycycline. **D.** Corresponding GAPDH loading control from mouse tumors.

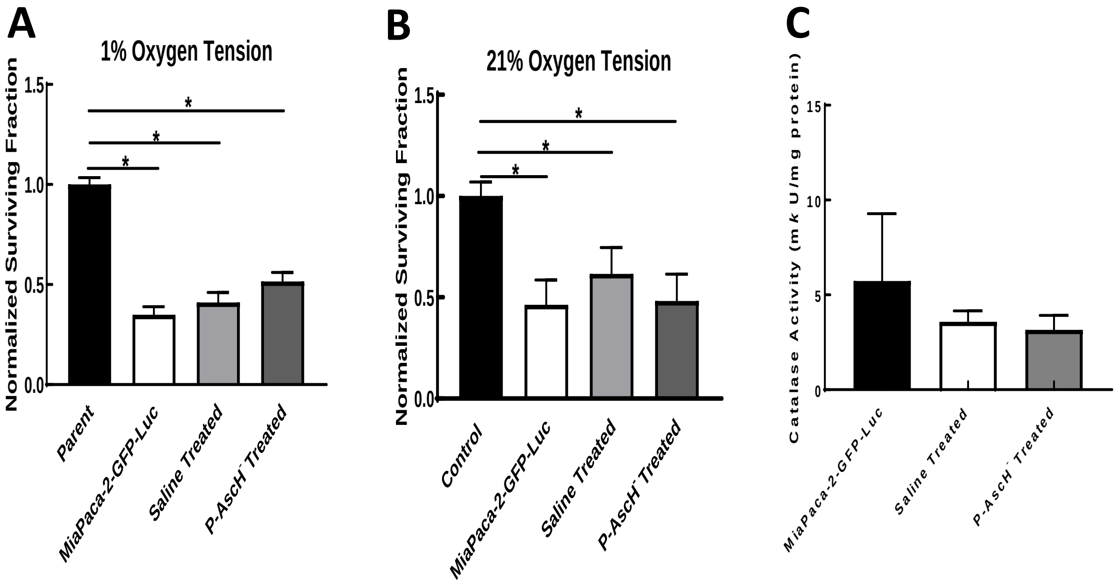


**Supplemental Figure 4. No changes in P-AscH$^-$ toxicity or catalase activity in metastatic disease.**

**A** and **B**. Liver specimens with visible metastatic lesions were used to isolate single tumor cells from both saline treated and P-AscH$^-$ treated mice. Cells were cultured at both 1% and 21% oxygen tension following confirmation of both GFP and luciferase expression after isolation. The stably expressing cell line (MIA PaCa-2-GFP-Luc) and isolated tumor cells from both saline treated and P-AscH$^-$ treated mice were incubated with 1 mM for 1 h and assessed for clonogenic cell survival. Colony formation was determined after 7-10 days. No differences were detected in clonogenic survival between the stably expressing cell line, saline treated, and P-AscH$^-$ treated isolated cells compared to the parental cell line. Surviving fraction was decreased with the addition of P-AscH$^-$ in the stably expressing cell line and isolated tumor cell lines compared to controls. Data represent normalized surviving fractions compared to controls ± SE (n = 3, * $p < 0.05$; one-way ANOVA with Bonferroni's multiple comparisons).

**C**. Isolated tumor cells from saline treated and P-AscH$^-$ treated mice where tested for catalase activity and compared to MIA PaCa-2-GFP-Luc cells. No differences were detected in catalase activity between stable expressing cells and saline or P-AscH$^-$ treated tumor cells. Data represent catalase activity in mK U\mg protein ± SE (n = 3, $p > 0.05$; one-way ANOVA with Bonferroni's multiple comparisons).
